# An ELISA Assay for Quantifying Monomeric C-Reactive Protein in Plasma

**DOI:** 10.3389/fimmu.2018.00511

**Published:** 2018-03-12

**Authors:** Lin Zhang, Hai-Yun Li, Wei Li, Zhi-Yuan Shen, Yin-Di Wang, Shang-Rong Ji, Yi Wu

**Affiliations:** ^1^MOE Key Laboratory of Environment and Genes Related to Diseases, School of Basic Medical Sciences, Xi’an Jiaotong University, Xi’an, China; ^2^MOE Key Laboratory of Cell Activities and Stress Adaptations, School of Life Sciences, Lanzhou University, Lanzhou, China; ^3^Ultrasound Department, The Second Hospital of Lanzhou University, Lanzhou, China

**Keywords:** inflammation, autoimmune diseases, urticaria, psoriasis, C-reactive protein, monomeric C-reactive protein

## Abstract

Native C-reactive protein (nCRP) is a non-specific marker of inflammation being claimed as a bystander in several chronic disorders. Accumulating evidence indicates that nCRP dissociates to and acts primarily as the monomeric conformation (mCRP) at inflammatory loci. This suggests that mCRP may be a superior disease marker with improved specificity and clear causality to the underlying pathogenesis. However, the lack of a feasible assay to quantify mCRP in clinical samples precludes the assessment of that suggestion. Here we report the development of a sandwich ELISA assay for quantification of plasma mCRP using commercially available reagents. Our assay is reproducible and highly conformation-specific showing a reliable detection limit of 1 ng/mL. We further show that mCRP appears to be a better marker than nCRP in several skin-related autoimmune disorders. This assay thus provides a useful tool to examine the clinical significance and utility of mCRP.

## Introduction

Native C-reactive protein (nCRP) is a major human acute phase reactant that responds to tissue damage or infection by rapidly increasing its blood concentrations (1, 2). It thus has been widely used as a non-specific marker of inflammation in clinical practice. Moreover, a minor increase in the circulating level of nCRP, originally considered as non-pathological, is found to be associated with the risk and prognosis of several chronic inflammatory disorders, including cancer (3) and cardiovascular disease (4). However, those associations are usually moderate and are shown to be non-causal by large-scale genetic studies (5, 6). Therefore, it appears plausible that nCRP may simply be a bystander in these diseases (3, 4).

Native C-reactive protein is composed of five identical subunits that are induced to dissociate at inflammatory loci (7–11) upon encountering damaged membranes (12–21), amyloid aggregates (22), neutrophil extracellular traps (23), or acidic pH (24). The dissociated conformation is termed as monomeric CRP (mCRP) and could be the major actor in local inflammation (7–11). This argues that mCRP may be a marker more specific to the underlying pathological processes. Indeed, circulating or microparticle-bound mCRP has been shown to be a better diagnostic index than nCRP in myocardial infarction (20, 25) and peripheral artery disease (26). However, the homemade assays used in these studies prohibit broad evaluation of the above argument. To clear that hurdle, here we develop a highly specific ELISA assay to measure plasma levels of mCRP based on commercially available reagents.

## Materials and Methods

### Reagents

Native C-reactive protein (purity > 97%) purified from human ascites was purchased from the BindingSite (Birmingham, UK; catalog number: BP300.X; lot number: 361639 and 404353) and repurified with p-Aminophenyl Phosphoryl Choline Agarose (Thermo Fisher Scientific, Rockford, IL, USA; catalog number: 20307). mCRP was prepared by treatment of nCRP with 8-M urea-EDTA (27) or by recombinant expression and purification (28, 29). Our assay worked well for both forms of mCRP. Proteins were dialyzed to remove NaN_3_, and passed through Detoxi-Gel Columns (Thermo Fisher Scientific, Rockford, IL, USA; catalog number: 20344) to remove endotoxin when necessary. Mouse antihuman CRP Abs 1D6 and 3H12 were generated as described (30, 31).

### ELISA Assay Quantifying nCRP

The sheep antihuman CRP polyclonal antibody (BindingSite; catalog number: PC044; lot number: 352325, 076682) was immobilized onto microtiter wells (Corning, NY, USA; catalog number: 42592; lot number: 10917007) at 2.5 µg/mL in coating buffer (10-mM sodium carbonate/bicarbonate, pH 9.6) overnight at 4°C. All the following steps were conducted at 37°C. Wells were washed with TBS (10-mM Tris, 140-mM NaCl, 2-mM Ca, pH 7.4) containing 0.02% NP-40, and then blocked with 1% BSA in TBS (blocking buffer). Samples diluted in blocking buffer were added into wells for 1 h. Captured CRP was detected with 1D6 mAb (1:300 in blocking buffer) that specifically recognizes the native conformation and an HRP-labeled goat anti-mouse IgG (H + L) (1:20,000 in blocking buffer) (Jackson ImmunoResearch, West Grove, PA; catalog number: 115-035-003; lot number: 125229). Wells were developed with TMB (Sigma-Aldrich; catalog number: T2885; lot number: WXBC2414V) and stopped with 1-M H_2_SO_4_. OD570 and OD450 nm were measured with a microplate reader. The OD value of each sample was calculated as OD450–OD570 nm. 100-µL volume was used at all incubation steps, while 300-µL volume was used for washing after each incubation step.

### ELISA Assay Quantifying mCRP

**Figure 1:**
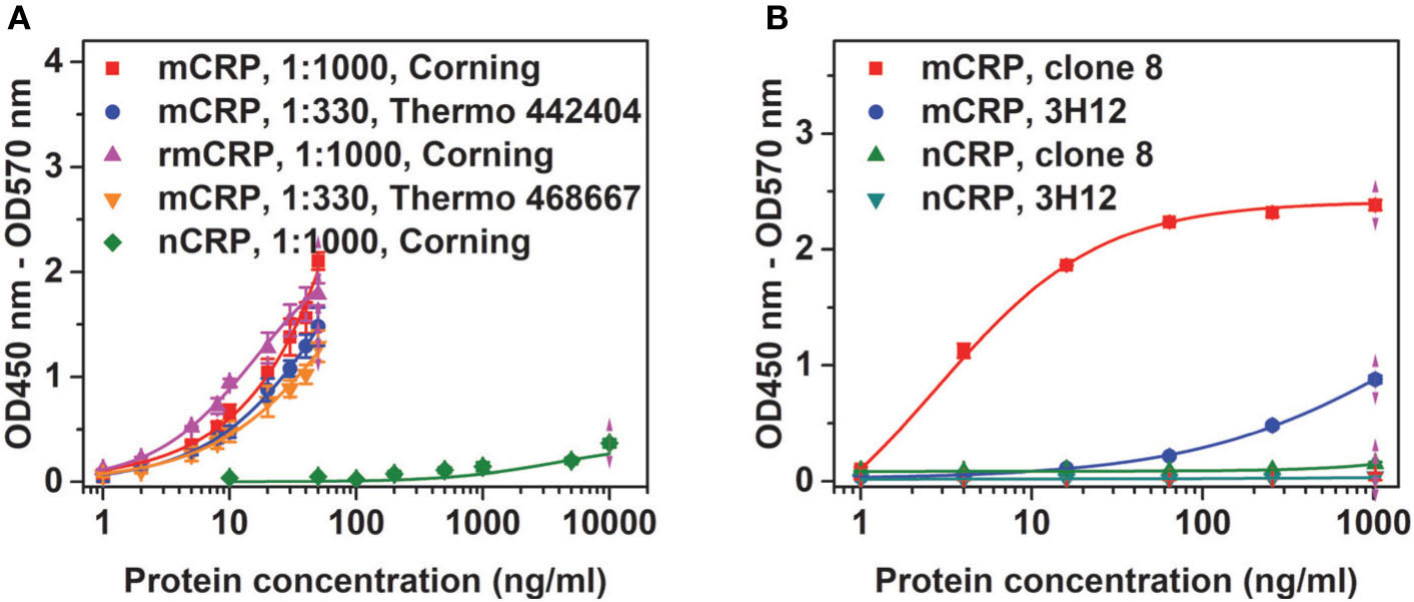
Conformation specificity of the assay. **(A)** Native C-reactive protein (nCRP), monomeric CRP (mCRP), or recombinant mCRP (rmCRP) at the indicated concentrations were added to the indicated wells coated with 1:1,000 or 1:330 CRP-8 mAb, and the captured proteins were detected with a polyclonal CRP antibody from the BindingSite. **(B)** nCRP or mCRP at the indicated concentrations were added to wells (Thermo Fisher; catalog number: 442404) coated with 1:330 CRP-8 (clone 8) or 1:200 3H12 mAbs, and the captured proteins were detected with a polyclonal CRP antibody from the BindingSite.

The mouse antihuman CRP mAb CRP-8 (Sigma-Aldrich, St. Louis, MO, USA; catalog number: C1688; lot number: 025M4863V) was immobilized onto microtiter wells (Thermo Fisher Scientific; catalog number: 468667, 442404; lot number: 148860, 148034; Corning, NY, USA; catalog number: 42592; lot number: 10917007) at 1:1,000 in coating buffer (10-mM sodium carbonate/bicarbonate, pH 9.6) overnight at 4°C. The performance of the assay was comparable regardless of the type of microtiter well used. 3H12 (1:200) was also immobilized as the capture antibody to compare its performance with that of CRP-8 as shown in Figure 1B. All the following steps were conducted at 37°C. Wells were washed with TBS containing 0.02% NP-40, and then blocked with 1% BSA in TBS (blocking buffer). TBS was made of 10-mM Trizma base (Sigma; catalog number: V900483), 140-mM NaCl (Amresco, Solon, OH, USA; catalog number: X190), and 2-mM CaCl_2_ (Sinopharm, Shanghai; catalog number: 10005861) in ultrapure water (>18.2 MΩ.cm) with pH adjusted to 7.4. Samples diluted in blocking buffer were added into wells for 1 h. Captured mCRP was detected with a sheep antihuman CRP polyclonal antibody (1:2,000 in blocking buffer) (BindingSite; catalog number: PC044; lot number: 352325, 076682) and an HRP-labeled donkey anti-sheep IgG (H + L) from Abcam (1:10,000 in blocking buffer) (Cambridge, UK; catalog number: ab6900; lot number: GR272029-6) or from Abbkine (1:20,000 in blocking buffer) (Wuhan, China; catalog number: A21060-1; lot number: ATQMA0601, ATQJN0701). Wells were developed with TMB and stopped with 1-M H_2_SO_4_. OD570 and OD450 nm were measured with a microplate reader. The OD value of each sample was calculated as OD450–OD570 nm. 100-µL volume was used at all incubation steps, while 300-µL volume was used for washing after each incubation step. Plasma samples were obtained from the First (urticaria) and Second Affiliated Hospitals (eczema and psoriasis) of Xi’an Jiaotong University. Informed consents for blood sampling were signed by all participants, and the research was in compliance with the Declaration of Helsinki and approved by the local ethical committee.

### Statistical Analysis

Data were presented as mean ± SEM. Statistical analysis was performed by one-way ANOVA, Kruskal–Wallis ANOVA or Kolmogorov–Smironv tests as appropriate. Differences were considered significant at values of *p* < 0.05.

## Results and Discussion

Sandwich ELISA is a convenient yet reliable assay for laboratory quantification of antigens in clinical samples without demanding equipment requirements. In case of measuring mCRP, the key to the success is to identify an appropriate pair of capture and detection antibodies. Such an antibody pair should be highly specific and sensitive to accurately quantify low level of mCRP [probably in ng/mL (25)] in the background of high level nCRP (usually in µg/mL). Polyclonal antibodies can bind both nCRP and mCRP, and therefore may not represent suitable candidates for being the capture antibody. Indeed, most screened pairs with polyclonal capture antibodies worked well for purified mCRP, but all failed in reconstituted mixtures or clinical samples containing both nCRP and mCRP (not shown).

**Figure 2:**
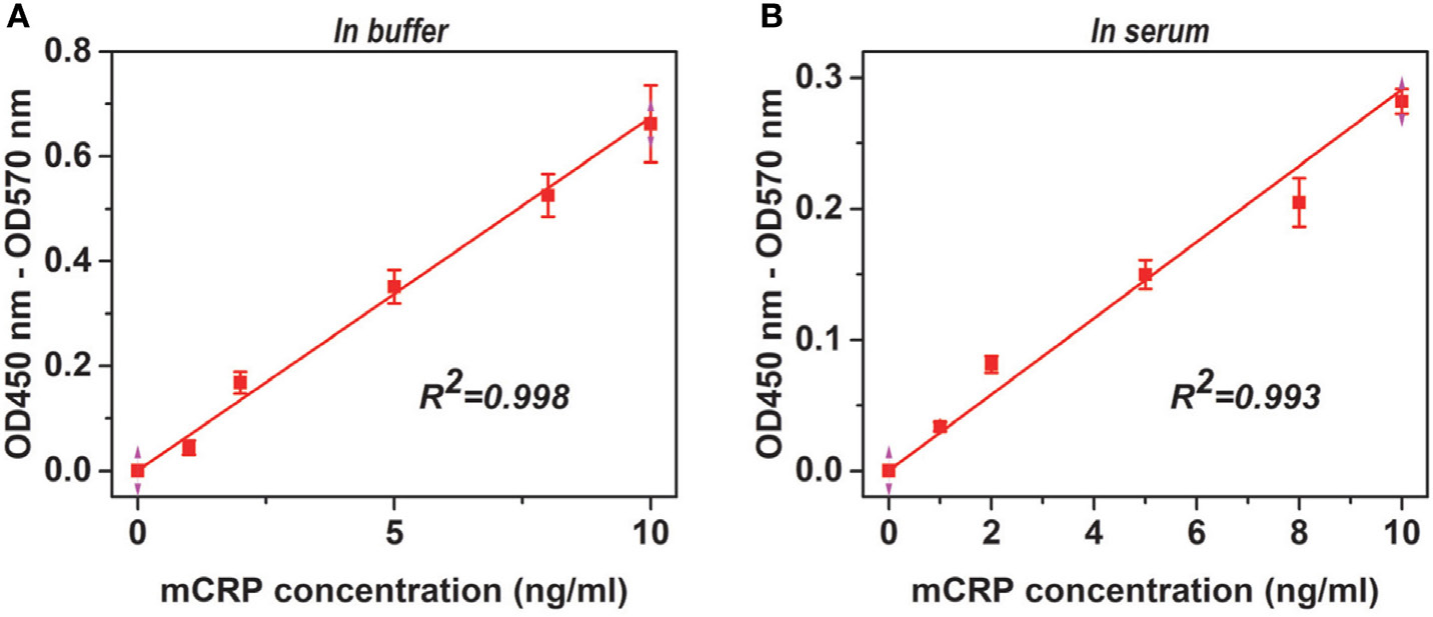
Adaptability of the assay. Monomeric CRP (mCRP) at the indicated concentrations in TBS buffer **(A)** or reference human sera (1:100) **(B)** were added to wells (Corning) coated with 1:1,000 CRP-8 mAb (clone 8), and the captured proteins were detected with a polyclonal CRP antibody from the BindingSite. The endogenous mCRP concentrations in the reference human sera were <10 ng/mL and therefore was undetectable following 1:100 dilution. The Pearson’s correlation coefficients (*R*) were also indicated.

For screened pairs with monoclonal capture antibodies, those using polyclonal detection antibodies performed better. A monoclonal capture antibody from Sigma (CRP-8) and a polyclonal detection antibody from BindingSite emerged as the best choice. This pair was highly selective, reaching half maximal signals for mCRP at ~20 ng/mL but generating only background signals for nCRP at 1 µg/mL (Figure 1A). The performance of CRP-8 as the capture antibody was even superior than that of 3H12, an established mAb of mCRP (30) (Figure 1B). This assay could reliably report mCRP as low as 1 ng/mL in both purified (Figure 2A) and complex samples (Figure 2B). Its robustness was further validated by using different batches of reagents and microtiter wells, by performing in laboratories at different cities by distinct colleagues over months.

**Figure 3:**
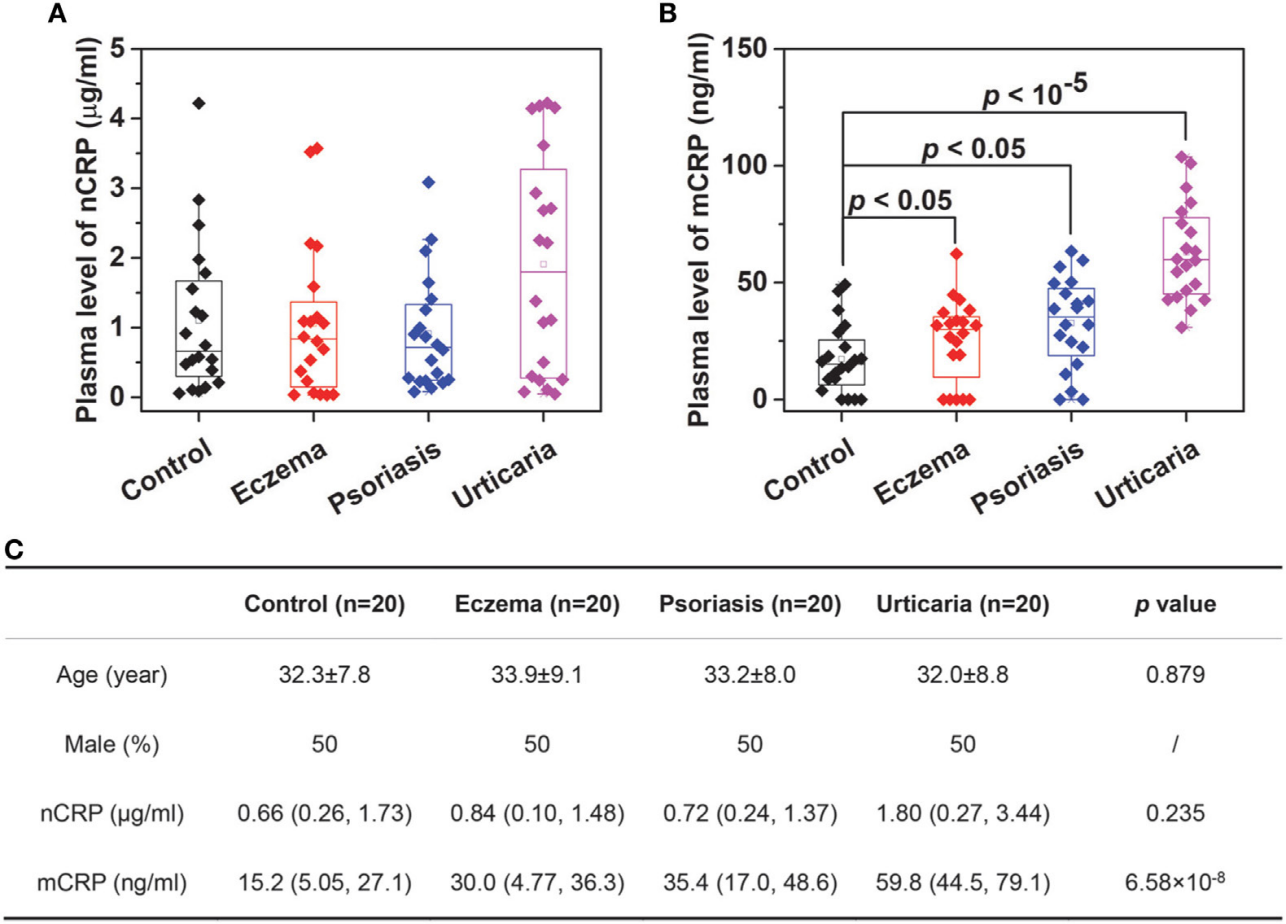
Determination of monomeric CRP (mCRP) levels in clinical samples. The plasma levels of native C-reactive protein (nCRP) **(A)** and mCRP **(B)** were determined in healthy controls (*n* = 20) and patients with eczema (*n* = 20), psoriasis (*n* = 20), and urticaria (*n* = 20) using Corning microtiter wells coated with 1:1,000 CRP-8. **(C)** The summarized results.

We next determined plasma levels of nCRP and mCRP in healthy controls and patients with skin-related autoimmune disorders, including eczema, psoriasis and urticaria (Figure 3). The levels of nCRP in patients did not differ significantly from that in controls (Figure 3A), whereas the levels of mCRP were significantly higher in patients than in controls (Figure 3B). Moreover, though mCRP was increased in all three disorders, the extents of increase differed significantly with the strongest increase observed in patients with active urticaria. These results suggest that mCRP is not only more sensitive to local status of inflammation but may also be specific to the underlying pathogenesis. Therefore, large-scale investigations and thorough analysis are warranted to establish the clinical significance of mCRP in diagnosis and prognosis, and the assay developed herein provides a means for that purpose.

## Ethics Statement

The study was approved by the Ethics Committee of Xi’an Jiaotong University. Informed consents for blood sampling were signed by all of the participants.

## Author Contributions

YW and S-RJ designed the research. LZ, H-YL, WL, Z-YS, and Y-DW performed the research. YW, S-RJ, LZ, and H-YL analyzed the data and wrote the paper. All authors reviewed the results and approved the final version of the manuscript.

## Conflict of Interest Statement

The authors declare that the research was conducted in the absence of any commercial or financial relationships that could be construed as a potential conflict of interest.
